# Point‐of‐Care Kidney Ultrasonography in Pediatric Nephrology: Current Evidence and Clinical Relevance

**DOI:** 10.1155/ijne/7767952

**Published:** 2026-06-14

**Authors:** Mahboube Bahroudi, Ali Asadifar, Mastaneh Moghtaderi

**Affiliations:** ^1^ Pediatric Chronic Kidney Disease Research Center, Gene, Cell & Tissue Research Institute, Children’s Medical Center, Tehran University of Medical Sciences, Tehran, Iran, tums.ac.ir

**Keywords:** diagnostic imaging, kidney ultrasonography, pediatric nephrology, point-of-care ultrasonography

## Abstract

Point‐of‐care ultrasonography (POCUS) has revolutionized pediatric healthcare by offering immediate, bedside diagnostic capabilities that enhance clinical decision‐making and patient management. This comprehensive review examines the impact and necessity of POCUS in assessing renal conditions in children. By synthesizing current literature, clinical applications, diagnostic accuracy, and patient outcomes associated with POCUS, this paper highlights its essential role in pediatric nephrology. Additionally, the integration of POCUS into clinical practice, requisite training protocols, and future technological advancements are discussed. The review concludes that POCUS is an indispensable tool in pediatric kidney assessment, improving diagnostic precision, reducing the need for more invasive procedures, and enhancing overall patient outcomes.

## 1. Introduction

Point‐of‐care ultrasonography (POCUS) has become an increasingly important bedside imaging modality in pediatric care. It enables real‐time clinical assessment without exposure to ionizing radiation. Portability, rapid availability, and the noninvasive nature of POCUS have made it particularly valuable in children [1–3]. While POCUS is now widely integrated into emergency medicine, critical care, and general pediatrics [4], its role in pediatric nephrology is of particular clinical relevance due to the chronic and evolving nature of kidney diseases in childhood.

Children with kidney disorders frequently require serial imaging for diagnosis, monitoring of disease progression, and guidance of interventions. Congenital anomalies of the kidney and urinary tract, hydronephrosis, acute kidney injury, and chronic kidney disease (CKD) represent major contributors to pediatric morbidity and long‐term renal impairment [5]. Early recognition of structural and functional abnormalities and longitudinal imaging follow‐up are central to preserving renal function over time. In this context, bedside kidney ultrasonography offers a practical adjunct to clinical assessment by enabling timely visualization of renal morphology and the urinary tract during routine patient encounters [6–8].

Although POCUS has been extensively incorporated into adult nephrology practice, its pediatric implementation has been more variable. Differences in patient size, cooperation, and developmental anatomy, as well as limited access to pediatric‐focused training pathways, have contributed to heterogeneous uptake across institutions [3, 6]. Nevertheless, advances in portable ultrasound technology and expanding educational initiatives are facilitating broader integration of kidney‐focused POCUS into pediatric renal care. These developments underscore the need for a focused synthesis of the current evidence supporting POCUS in pediatric renal care.

Accordingly, this narrative review aims to consolidate current evidence on the clinical applications, diagnostic performance, and practical integration of POCUS in pediatric nephrology. In contrast to prior literature that has largely focused on specific clinical settings, such as in pediatric intensive care units [9], this review offers a kidney‐focused, evidence‐based synthesis encompassing discrete renal indications, comparative diagnostic performance, and longitudinal applications across acute and CKD as a novel scope. By integrating current data with practical guidance on implementation, training, and future technological developments, this review provides clinicians and educators with a concise, clinically relevant framework for optimizing the use of POCUS in pediatric nephrology and facilitating its effective incorporation into routine kidney care pathways.

## 2. Methods

This study was conducted as a narrative review aimed at synthesizing current evidence on the clinical applications and relevance of point‐of‐care kidney ultrasonography (POCUS) in pediatric nephrology. A comprehensive literature search was performed to identify relevant publications. Electronic searches were conducted in PubMed/MEDLINE, Web of Science, Scopus, and Google Scholar for articles published between 2000 and 2025. The search strategy used combinations of the following keywords and Medical Subject Headings (MeSH) where applicable: *“point-of-care ultrasound”, “POCUS”, “renal ultrasound”, “kidney ultrasonography”, “pediatric nephrology”, “hydronephrosis”, “chronic kidney disease”, “renal trauma”,* and *“kidney imaging in children”*. Eligible articles included original research studies, reviews, and clinical guidelines that addressed the use of POCUS or bedside renal ultrasonography in pediatric populations. Studies focusing exclusively on adult patients or nonrenal ultrasound applications were excluded unless they provided relevant contextual or methodological insights applicable to pediatric nephrology. Additional references were identified through manual screening of reference lists from key articles to ensure comprehensive coverage of the topic. Selected studies were reviewed and integrated based on their clinical relevance, methodological rigor, and contribution to understanding the role of POCUS in pediatric kidney disease.

## 3. Result

### 3.1. Principles and Techniques of Point‐of‐Care Kidney Ultrasonography

POCUS utilizes high‐frequency transducers to obtain detailed images of renal structures at the bedside. Selecting the appropriate transducer is critical for optimizing image quality; linear transducers with high frequencies are typically used for superficial structures, while lower‐frequency convex or phased‐array transducers are employed for deeper areas [10]. Proficiency in scanning techniques, such as longitudinal and transverse scans, is crucial for evaluating kidney size, shape, cortical thickness, and structural abnormalities [11]. Doppler ultrasound is used to evaluate renal blood flow and detect vascular issues such as renal artery stenosis or thrombosis. Additionally, POCUS can estimate bladder volume and evaluate post‐void residual, assisting in the diagnosis and management of conditions such as vesicoureteral reflux (VUR) and urinary retention [12].

The noninvasive nature of POCUS, combined with the absence of ionizing radiation, makes it particularly advantageous for use in children, allowing for repeated assessments without cumulative risks [3]. Immediate diagnostic information facilitates prompt clinical decision‐making, enhancing patient care quality. However, POCUS is inherently operator‐dependent, with accuracy contingent on the clinician’s expertise and experience. Factors such as patient cooperation, body habitus, and the presence of overlying gas or structures can influence image quality. To ensure consistent diagnostic performance, continuous training and adherence to standardized protocols are essential. Establishing comprehensive training programs and certification processes helps maintain high standards of practice and optimizes POCUS’s effectiveness in pediatric care [13].

### 3.2. Clinical Applications of POCUS in Pediatric Kidney Assessment

POCUS has a broad spectrum of applications in assessing renal conditions in children. Its versatility allows for the evaluation of both acute and CKD, the detection of structural anomalies, and the guidance of interventional procedures and follow‐up of kidney problems. The next parts of this document investigate the main clinical roles of POCUS in pediatric renal assessments [14].

### 3.3. Detection of Hydronephrosis

**FIGURE 1:**
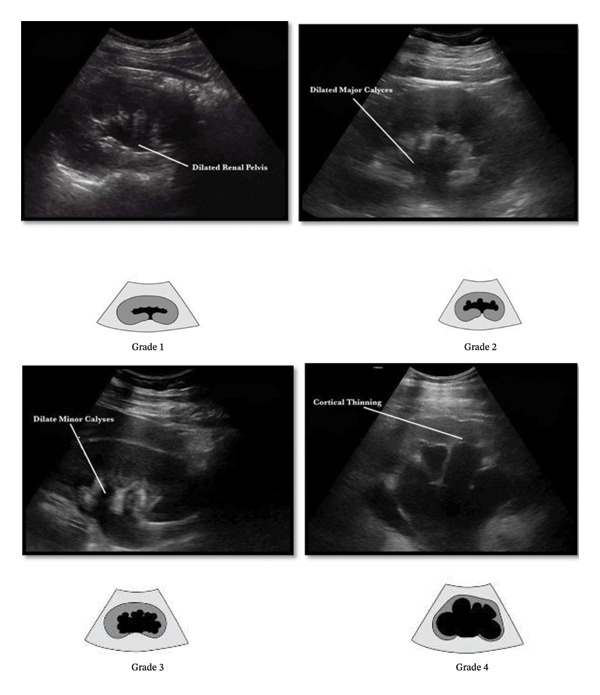
Society for Fetal Urology Grading System. This classification is based on the appearance of the renal pelvis, calyces, and renal parenchyma. Grade 0: no hydronephrosis; intact central renal complex. Grade 1: only dilated renal pelvis; there is some fluid in the renal pelvis. Grade 2: dilated renal pelvis and a few calyces are visible. Grade 3: all the calyces are dilated. Grade 4: further dilation of renal pelvis and calyces, with thin renal parenchyma. (Modified from Baskin LS. Prenatal hydronephrosis. In: Baskin LS, Kogan B, Duckett J, editors. Handbook of Pediatric Urology. Philadelphia: Lippincott‐Raven; 1997. p. 15.281).

**FIGURE 2:**
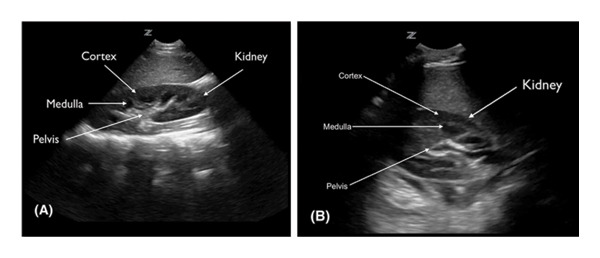
Normal Sonographic Appearance of a Pediatric Patient’s Right Kidney at (A) longitudinal and (B) transverse plane.

Hydronephrosis, characterized by the dilation of the renal pelvis and calyces, is a prevalent renal anomaly in pediatrics caused by factors such as congenital urinary tract obstructions, VUR, and ureteropelvic junction (UPJ) obstruction. POCUS is crucial for the early detection and grading of hydronephrosis using the Society for Fetal Urology (SFU) grading system, which classifies the condition into four grades based on the extent of pelvis dilation and calyceal involvement [15, 16]. Early identification through POCUS enables the recognition of children at risk for renal damage and allows for close monitoring of disease progression [17]. The first step for evaluating hydronephrosis is voiding cystourethrography (VCUG). When there is not any reflux or the bladder is normal, significant hydronephrosis detected by POCUS may indicate UPJ obstruction, necessitating more evaluation with diuretic renography. Sometimes surgical intervention is needed to prevent renal scarring and loss of function. Studies have demonstrated that POCUS possesses high sensitivity and specificity in detecting hydronephrosis and that trained pediatricians using POCUS achieve diagnostic accuracy comparable to radiology‐performed ultrasonography [7]. Additionally, POCUS assists in guiding interventional procedures, such as the placement of nephrostomy tubes, ensuring precise localization and reducing procedural complications [18] (Figures 1 and 2).

The grading of hydronephrosis using POCUS can be described as follows [19]: Grade I represents minor dilation of the renal pelvis without involvement of the calyces. Grade II indicates moderate dilation with mild calyceal involvement. Grade III is characterized by marked dilation accompanied by significant calyceal involvement. Grade IV denotes severe dilation with associated thinning of the renal cortex.

### 3.4. Evaluation of Renal Masses and Cysts

Renal masses and cysts, though uncommon in children, require careful evaluation due to their potential malignancy and the necessity for appropriate management. POCUS acts as an initial diagnostic tool to differentiate between benign and malignant renal lesions, guiding further diagnostic and therapeutic actions. The renal sonographic features in Autosomal Recessive Polycystic Kidney Disease (ARPKD) subjects included a normal‐sized kidney with no cysts or multiple small cysts, increased cortical echogenicity, and loss of corticomedullary differentiation. In Autosomal Dominant Polycystic Kidney Disease (ADPKD) patients, sonographic features include a large kidney with few or multiple cysts of different sizes [20]. POCUS distinguishes solid masses from cystic lesions by assessing echogenicity, margins, septations, and calcifications. Simple cysts appear anechoic with well‐defined margins, while solid masses may show hyperechoic or isoechoic areas with irregular borders and internal vascularity on Doppler imaging.

The occurrence of renal tumors, particularly Wilms’ tumor, in the pediatric population highlights the importance of prompt and accurate imaging for both diagnosis and staging. POCUS can detect suspicious renal masses, thereby facilitating timely referral for confirmatory imaging with MRI or contrast‐enhanced CT to establish a definitive diagnosis and guide treatment planning [21]. For known benign cysts or masses, POCUS offers a convenient method for routine monitoring, allowing clinicians to track changes without exposing children to repeated radiation from other imaging modalities [22].

**FIGURE 3:**
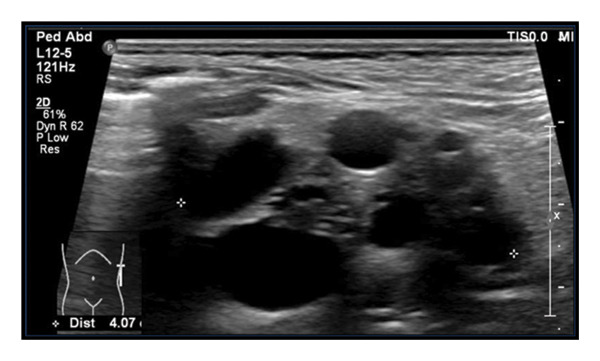
Renal Cysts. An 8‐month‐old boy with a multicystic dysplastic kidney. On ultrasound, multiple cysts are present, and no normal renal tissue is seen.

Studies have shown that POCUS is highly accurate in identifying features suggestive of malignancy, with a study demonstrating that it effectively identified malignant characteristics in pediatric renal masses, facilitating early referrals for oncological evaluation and management [23] (Figure 3).

### 3.5. Assessment of Renal Trauma

**FIGURE 4:**
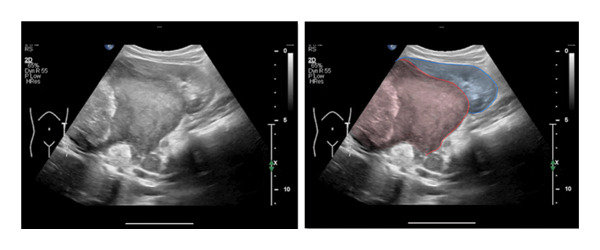
Renal Mass. Three‐years‐old girl with a palpable left flank mass. Ultrasound shows a mass (outlined in red) originating from the left kidney (blue). The mass is very inhomogeneous with calcifications and is compatible with a neuroblastoma.

Renal injuries, though rare, can result from blunt or penetrating trauma, potentially leading to significant morbidity if not quickly identified and managed. Pediatric patients are particularly vulnerable due to their unique anatomical and physiological characteristics. In these cases, POCUS is a crucial tool for both initial assessment and ongoing management [24]. As part of the extended focused assessment with sonography for trauma (eFAST), POCUS quickly detects free fluid in the peritoneal or retroperitoneal spaces, indicating possible organ injury [24, 25]. Specifically for renal trauma, POCUS can identify signs like perinephric hematomas, renal lacerations, or urinary extravasation. In emergency settings where time is critical, POCUS provides a quick assessment of injury severity, enabling timely decisions for surgical intervention in severe cases. Renal injuries such as lacerations appear as hypoenhancing areas, which are often wedge‐shaped and are highly suggestive of active bleeding. This helps minimize the risk of complications such as continuing renal hemorrhage or acute kidney injury, although CT is the mainstay for diagnosing renal injuries [26]. For pediatric patients with renal trauma, POCUS also allows for ongoing monitoring, especially in conservative cases, ensuring proper healing and preventing complications like persistent hematomas or pseudoaneurysms. Studies have demonstrated POCUS’s high sensitivity in detecting renal injuries [27] (Figure 4).

### 3.6. Monitoring CKD

CKD in children is a progressive condition that demands careful monitoring to prevent complications and slow disease progression. POCUS provides a noninvasive, efficient way to regularly assess renal size, parenchymal changes, and complications like hypertension‐induced nephropathy or secondary hyperparathyroidism [5, 28]. In CKD, kidneys often shrink over time, a condition called renal hypoplasia or atrophy. POCUS allows for accurate measurement of kidney size and cortical thickness, both of which are crucial indicators of disease severity and progression. Monitoring these changes helps guide treatment adjustments, including the timing of interventions like renal transplantation. POCUS also plays a key role in identifying CKD‐related complications, such as renal cysts, medullary sponge kidney, and increased renal echogenicity, which may signal interstitial fibrosis or glomerulosclerosis [29, 30]. Additionally, Doppler ultrasound can evaluate renal blood flow, offering insights into the hemodynamic changes that occur in CKD, including hypertensive nephropathy. In certain cases, POCUS can also guide interventions, such as the placement of hemodialysis access or peritoneal dialysis catheters, helping ensure proper positioning and reducing procedural risks [31, 32]. By providing regular, real‐time data, POCUS supports comprehensive CKD management, allowing for early detection of worsening renal function or emerging complications. This proactive monitoring aids in adjusting treatment plans, including medication, dietary restrictions, and other interventions, ultimately improving patient outcomes and quality of life. Research supports the value of routine POCUS in pediatric CKD care [8].

### 3.7. Diagnostic Accuracy and Comparative Effectiveness

POCUS has been widely studied and proven reliable for detecting pediatric renal conditions. It offers comparable diagnostic accuracy to traditional imaging methods, such as radiologist‐performed ultrasonography, MRI, and CT scans, while providing the added benefits of bedside availability and immediacy [33]. POCUS has demonstrated high sensitivity and specificity for conditions like hydronephrosis, renal masses and cysts, and trauma‐induced injuries [7]. Unlike traditional imaging, which requires patient transport and scheduling delays, POCUS can be performed immediately at the bedside, offering rapid diagnostic insights that can significantly influence patient management, particularly in emergency settings [34]. Additionally, POCUS is cost‐effective, reducing the need for more expensive imaging techniques and minimizing healthcare expenditures. Its portability and ease of use make it suitable for diverse settings without major infrastructure investment [35]. Some studies have shown that POCUS achieves diagnostic accuracy rates compared to conventional ultrasonography, making it a reliable tool, particularly in resource‐limited or urgent situations [36]. However, POCUS has limitations, including operator dependency and its role as an initial screening tool. More complex renal pathologies may require additional imaging for a comprehensive diagnosis [37]. Meta‐analyses and systematic reviews support its use in pediatric nephrology with proper training and quality assurance [38, 39].

### 3.8. Training and Competency in Pediatric POCUS

Effective use of POCUS in pediatric nephrology depends heavily on the competency of healthcare providers. Comprehensive training and standardized competency assessments are crucial for ensuring accurate image acquisition and interpretation [37]. Training in pediatric POCUS involves a multifaceted approach combining theoretical education, practical skills, and clinical experience. It starts with basic ultrasound physics and anatomy, covering ultrasound wave propagation and pediatric renal anatomy fundamental for producing and interpreting quality images. Practical training follows, focusing on scanning techniques like longitudinal and transverse orientations, Doppler use, and pediatric bladder scanning protocols. Understanding the clinical applications of POCUS is equally important. Trainees learn to use POCUS for detecting hydronephrosis, assessing renal trauma, and monitoring acute and CKD. These skills ensure they can effectively apply POCUS across various pediatric scenarios, enhancing its value in nephrology [40, 41].

Continuous education is vital, given the advancing technology and changing clinical uses of POCUS. Advanced training, refresher courses, and workshops help maintain and enhance skills. [42]. Incorporating POCUS training into medical education fosters early familiarity and competence, encouraging its routine adoption in clinical practice. This integration provides future clinicians with essential skills and highlights ultrasound’s significance in modern medicine [34]. Also, challenges remain, such as limited access to resources, variable training quality, and the demands on medical education schedules. Overcoming these barriers requires institutional support, investment in infrastructure, and standardized curricula [43].

In summary, comprehensive POCUS training is essential for developing skilled practitioners. By investing in education and tackling existing challenges, the medical community can enhance provider proficiency, leading to better diagnostic accuracy and patient outcomes in pediatric care (Table 1).

**TABLE 1:** Recommendations for enhancing training.

Standardization of training protocols	Developing universally accepted training guidelines ensures consistency in education quality across different institutions
Simulation‐Based Training	Utilizing ultrasound simulators and virtual reality platforms can enhance hands‐on learning without the need for live patient interactions, thereby broadening training opportunities
Mentorship and Supervision	Pairing trainees with experienced POCUS practitioners facilitates knowledge transfer, provides real‐time feedback, and fosters a culture of continuous learning and improvement

### 3.9. Integration Into Clinical Practice

Successfully integrating POCUS into pediatric nephrology requires careful planning, collaboration among healthcare teams, and modifications to ensure it becomes a natural part of patient care. Developing clear protocols is essential; these guidelines should define when and how to use POCUS, as well as establish documentation standards. For instance, protocols might specify using POCUS to screen for hydronephrosis in infants or to assess suspected renal trauma in emergencies. Consistent application of these protocols ensures that POCUS enhances rather than disrupts standard care practices [44, 45].

Investing in high‐quality, portable ultrasound devices are crucial for effective POCUS implementation. When selecting devices, factors such as portability, battery life, image quality, and user‐friendliness should be considered. Additionally, regular maintenance and updates of both hardware and software are necessary to keep the equipment reliable and functional. Successful integration often involves collaboration across various departments, including radiology, nephrology, emergency medicine, and intensive care [38, 46]. Radiology teams can provide expertise in ultrasound imaging, help develop protocols, and ensure quality control, while nephrology specialists can identify specific clinical needs to ensure that POCUS addresses relevant patient care goals [47].

Maintaining high standards through quality assurance is vital for the effective use of POCUS. Regular examinations, peer reviews, and feedback systems help identify areas for improvement and ensure adherence to established protocols [48]. Continuous improvement initiatives, such as updating training programs based on new evidence and technological advancements, further enhance the integration of POCUS into clinical practice [49]. Educating patients and their families about POCUS is also crucial, especially in pediatric settings where trust and comfort are key. Clear communication about the noninvasive nature of POCUS, its diagnostic purposes, and what to expect during the procedure can improve patient experience and cooperation, creating a more supportive clinical environment [50].

Despite its benefits, integrating POCUS faces several challenges. Resistance to change among healthcare providers, who may prefer traditional imaging methods, can delay adoption. Limited training resources and financial constraints for purchasing ultrasound devices also pose significant obstacles. Additionally, inconsistent institutional support can lead to uneven implementation across different healthcare settings. Addressing these barriers requires comprehensive strategies, including securing administrative support to prioritize POCUS, allocating budgets for equipment and training, and supporting the benefits of POCUS through evidence‐based practices. By tackling these challenges, healthcare institutions can fully leverage POCUS, enhancing patient care and diagnostic accuracy in pediatric nephrology [47, 51].

### 3.10. Benefits and Limitations of POCUS in Pediatric Nephrology

POCUS is becoming an increasingly valuable tool in pediatric nephrology, offering numerous benefits while also having some limitations that need to be considered. Its primary benefit is the ability to provide quick, radiation‐free imaging, which is especially important for children who are more susceptible to the harmful effects of radiation from procedures like CT scans. Unlike other imaging methods, POCUS does not expose children to ionizing radiation, making it a safer alternative, particularly when frequent imaging is required for chronic conditions [52, 53].

In addition to being safer, POCUS is also cost‐effective. Delivering immediate results reduces the need for more expensive imaging techniques and can help simplify care by guiding clinical decisions without the delays typically associated with traditional imaging [35]. This ability to provide real‐time feedback is crucial in fast‐paced settings, such as emergency rooms or intensive care units, where rapid decision‐making can significantly impact patient outcomes [54]. Furthermore, POCUS is a flexible tool that can be used repeatedly without causing harm to the patient, making it invaluable for ongoing monitoring, especially in children with long‐term renal conditions [3]. Another key advantage of POCUS is its portability, it can be easily moved to various clinical settings, even in rural or resource‐limited areas, offering support in situations where traditional imaging might not be feasible [55]. POCUS also plays a pivotal role in interventional procedures like nephrostomy tube placements and renal biopsies, helping clinicians navigate these delicate tasks more safely and accurately [56].

Despite these clear benefits, POCUS does have limitations. One of the most significant is the fact that its accuracy depends heavily on the operator’s skill and experience. Inconsistent training and proficiency among users can lead to variable results, meaning that diagnostic accuracy may not always be reliable [33, 57].

Moreover, while POCUS is excellent for initial assessments and monitoring, it cannot always provide the detailed information needed for comprehensive diagnoses. In more complex cases, advanced imaging techniques like CT or MRI are often necessary to confirm or further explore findings. The need for ongoing training and regular competency checks is also a challenge, as clinicians need to maintain their skills over time, which can be resource‐intensive. Furthermore, since there are currently no standardized protocols for POCUS across institutions, practices can contrast, leading to inconsistency in diagnostic approaches and outcomes [58, 59].

### 3.11. Future Directions and Innovations

The future of POCUS in pediatric nephrology is highly promising, with several technological and practice‐based advancements poised to enhance its clinical utility. In adult medicine—including adult nephrology—POCUS is already well integrated into routine practice and has demonstrated substantial benefits in acute care, dialysis access evaluation, and CKD monitoring. These successful adult applications provide a valuable model for pediatrics, illustrating how evidence‐based protocols, structured training programs, and multidisciplinary adoption can accelerate pediatric integration. Innovations such as incorporating artificial intelligence (AI) into ultrasound machines can improve image acquisition and interpretation, reducing operator dependency and boosting diagnostic accuracy [60]. Enhanced Doppler modalities, including superb microvascular imaging (SMI), allow detailed assessment of renal blood flow—critical for conditions like renal artery stenosis and acute kidney injury [61]. Device miniaturization is making POCUS even more portable and accessible, enabling use in diverse clinical environments, including remote and resource‐limited settings. Similarly, advancements in 3D and 4D ultrasonography offer enriched anatomical visualization, supporting the assessment of complex renal anomalies and improving clinical decision‐making [62, 63]. Tele‐ultrasonography facilitates real‐time remote consultation with specialists, benefiting regions with limited expert availability [64]. Contrast‐enhanced ultrasound (CEUS) further enhances visualization of the renal vasculature and assists in differentiating lesions [65]. Emerging systems capable of continuous kidney function monitoring hold promise for early intervention and truly personalized care, contributing to healthcare delivery that is more precise, proactive, and accessible [66].

## 4. Conclusion

Point‐of‐care kidney ultrasonography has established itself as an essential tool in pediatric nephrology, offering numerous benefits that enhance diagnostic accuracy, increase patient management, and improve clinical outcomes. Its noninvasive nature, coupled with the ability to provide immediate diagnostic information, makes POCUS an invaluable advantage in both acute and chronic renal care settings. The integration of POCUS into clinical practice, supported by comprehensive training programs and standardized protocols, ensures that healthcare providers can effectively utilize this technology to meet the diverse needs of pediatric patients. Despite innate limitations, such as operator dependency and variable image quality, the advantages of POCUS in terms of safety, cost‐effectiveness, and accessibility underscore its necessity in modern pediatric nephrology. Ongoing advancements in ultrasound technology, including AI integration, enhanced Doppler techniques, and tele‐ultrasonography, promise to further augment the utility and effectiveness of POCUS, addressing current limitations and expanding its applications. As the amount of evidence in favor of POCUS progressively increases, its role in pediatric kidney assessment is set to become even more essential, developing a more active and accurate approach to renal care in children. To make the most of POCUS’s capabilities, sustained efforts in training, research, and technological innovation are essential, ensuring that it remains at the progressive of pediatric nephrology and continues to contribute to improved patient health and well‐being.

## Author Contributions

Mahboube Bahroudi: conceptualization, investigation, methodology, visualization, and writing–original draft; Ali Asadifar: conceptualization, investigation, methodology, visualization, and writing–review and editing; Mastaneh Moghtaderi: conceptualization, methodology, project administration, resources, supervision, validation, and writing–review and editing.

## Funding

This research received no specific grant from any funding agency, commercial, or not‐for‐profit sectors.

## Ethics Statement

This study is a narrative review based on previously published literature and did not involve human participants, animal experiments, or the collection of personal data. Therefore, ethics approval and informed consent were not required.

## Conflicts of Interest

The authors declare no conflicts of interest.

## Data Availability

The authors have nothing to report.
